# The effect of oral supplementation of Paricalcitol on C-reactive protein levels in chronic kidney disease patients: GRADE-assessed systematic review and dose-response meta-analysis of data from randomized controlled trials

**DOI:** 10.1186/s40360-024-00740-y

**Published:** 2024-02-23

**Authors:** Seyyed Mostafa Arabi, Mostafa Shahraki-Jazinaki, Mahla Chambari, Leila Sadat Bahrami, Sara Sabeti, Mohammaed Ibrahim Mohaildeen Gubari, Basil D. Roufogalis, Amirhossein Sahebkar

**Affiliations:** 1 Noncommunicable Diseases Research Center, Neyshabur University of Medical Sciences, Neyshabur, Iran; 2 Healthy Ageing Research Centre, Neyshabur University of Medical Sciences, Neyshabur, Iran; 3 Department of Nutrition, School of Medicine, Mashhad University of Medical Sciences, Mashhad, Iran; 4 Department of food science and nutrition, School of Medicine, Gonabad University of Medical Sciences, Gonabad, Iran; 5 Department of Family and Community Medicine, College of Medicine, University of Sulaimani, Kurdistan region of Iraq, Sulaimani, Iraq; 6 Discipline of Pharmacology, School of Medical Sciences, University of Sydney, Sydney, NSW Australia; 7 NICM Health Research Institute, Western Sydney University, Penrith, NSW Australia; 8 Biotechnology Research Center, Pharmaceutical Technology Institute, Mashhad University of Medical Sciences, Mashhad, Iran; 9 Applied Biomedical Research Center, Pharmaceutical Technology Institute, Mashhad University of Medical Sciences, Mashhad, Iran

**Keywords:** Chronic kidney disease, Paricalcitol, C-reactive protein, Meta-analysis, Systematic review

## Abstract

**Background:**

Previous studies investigating the effect of oral supplementation of paricalcitol on reactive protein levels in chronic kidney disease (CKD) patients reported inconsistent findings. In this systematic review and meta-analysis, we have analyzed and interpreted the results obtained from previous randomized clinical trials on the effect of paricalcitol on C-reactive protein in CKD patients in the literature.

**Methods:**

MEDLINE, SciVerse Scopus, and Clarivate Analytics Web of Science databases were searched until January 2023 and related articles were obtained through a careful screening process allowing extraction of required data from selected articles. The effect size was calculated using a random effect model and weighted mean differences (WMD) and 95% confidence intervals (CI). Heterogeneity among studies was evaluated using Cochran’s Q test and I^2^.

**Results:**

Amongst the 182 articles obtained from the initial search, 4 studies (6 arms) were finally included in the meta-analysis. Pooled analysis shows that C-reactive protein levels significantly decrease after oral supplementation with paricalcitol (WMD: -2.55 mg/L, 95% CI (-4.99 to -0.11; *P* = 0.04). The studies used in this meta-analysis showed significant heterogeneity (I^2^ = 66.3% and *P* = 0.01).

**Conclusion:**

Oral paricalcitol supplementation in CKD patients can significantly reduce C-reactive protein levels, which may prevent CKD progression.

**Supplementary Information:**

The online version contains supplementary material available at 10.1186/s40360-024-00740-y.

## Introduction

Chronic kidney disease (CKD) is defined as a condition in which the glomerular filtration rate is less than 60 mL/min/1.73 m^2^, for more than 3 months [1]. CKD, as a health-related problem, affects more than 10% of the global population [2]. Previous studies have reported a significant relationship between the progression of CKD and the occurrence of hyperlipidemia, anemia, malnutrition, bone metabolic disorders, and cardiovascular events [3, 4]. CKD, by changing vitamin D metabolism, leads to an increased prevalence of vitamin D deficiency in these patients compared to others [4]. The severity of vitamin D deficiency can vary depending on the stage of CKD and the presence of secondary hyperparathyroidism (SHPT) in these patients [5–7]. Supplementation with the active form or analogue of vitamin D in these patients can be recommended [8]. On the other hand, the progression of CKD has been closely related to the severity of inflammation and oxidative stress in these patients [3]. The chronic inflammatory condition that CKD patients experience has a primary role in their high morbidity and mortality rate. An inflammatory situation, often associated with uremia, can be identified by the determination of biochemical parameters such as CRP levels [9]. The C-reactive protein (CRP), first identified in 1930 by Tillet and Frances [10], is an acute-phase protein and a marker of systemic inflammation [11]. CRP is produced by hepatocytes and is a member of the pentraxin family and its levels in the blood are regulated by interleukin 1) IL-1( and 6 (IL-6), Tumor Necrosis Factor-alpha) TNF-alpha( and inflammatory cytokines [11]. C-reactive protein levels in most healthy people are usually less than 10 mg/dl [12]. Due to the high half-life of the C-reactive protein and the low cost of its evaluation, it is often used as an indicator of inflammation in clinical research [13]. Vitamin D, with its regulatory role in the production of inflammatory cytokines, is considered to be one of the most important factors modulating inflammation [14, 15]. Paricalcitol [19-nor-1, 25(OH) 2D2] is one of several vitamin D analogs developed to maintain the suppressive effect of calcitriol on parathyroid. Due to its low calcemic effect, paricalcitol is known as an effective and safe tool to control hyperparathyroidism [16]. By mimicking the function of calcitriol, paricalcitol binds to vitamin D receptors, regulates the expression of vitamin D-responsive genes, and finally prevents the release of parathyroid hormones [17]. Furthermore, the active forms of vitamin D are considered to have anti-inflammatory effects. For example, vitamin D reduces the production of TNF-α, T-helper type 1, interferons, and interleukins and suppresses the inflammatory reactions of macrophages [18]. Therefore, at present new active vitamin D compounds are being recommended due to differences in safety, and classical and non-classical activities such as anti-inflammatory effects and immune system modulation, with the expectation of preventing or reversing cardiovascular complications related to kidney diseases [19–21].

The present study aims to assess the effect of paricalcitol supplementation on C-reactive protein as a biomarker of inflammation. The impact of different dosages and duration of supplementation on CRP level is also evaluated.

## Methods

This review was conducted based on the guidelines of Prisma for systematic review and meta-analysis [22]. Before this, the protocol for conducting this systematic review and meta-analysis had been registered in the Prospero database with the registration number: CRD42023405139.

### Search strategy

The search strategy related to the objectives of this review was performed independently from the databases of MEDLINE, SciVerse Scopus, and Clarivate Analytics Web of Science up until January 2023 by two researchers M.ShJ; and SM.A. This study had no time and language limitations.

The search strategy of this review was designed using MeSH and non-MeSH keywords, which include: (“paricalcitol” OR “Zemplar” OR “vitamin D2” OR “1,25-dihydroxy ergocalciferol” AND “inflammation” OR “inflammatory markers” OR “CRP” OR “C-reactive protein” OR “hs-CRP” OR “High sensitive C-reactive protein” AND “RCTs” OR “randomized controlled trial”) The search strategy is provided in Supplementary Table 1.

To ensure that no eligible articles were missed, the references of the papers obtained from the initial search were also checked for relevant articles.

### Study selection

The articles obtained from the initial search were screened independently by two researchers M.ShJ and SM by examination of the article titles and abstracts to identify eligible studies.

In this review, the eligibility criteria for the studies to be included were determined based on the PICOS (Population, Intervention, Comparison, Outcomes, Study design) framework [23].

The eligibility criteria include (A) Population: CKD patients older than 18 years; (B) Intervention: supplementation with paricalcitol; (C) Comparison: placebo; (D) Outcomes: CRP level; (E) Study design: RCTs.

Exclusion criteria included (A) animal studies, (B) observational studies, reviews, letters to the editor, and short communications.

### Data extraction

Relevant data were independently extracted from eligible articles by two researchers, M.ShJ and SM.A. These data include the first author’s name, year of publication; country, study design, number of volunteers in intervention and control groups, mean age of participants in each group, average BMI of subjects in each group, type of intervention, a daily dose of paricalcitol, duration of the study, mean and SD of outcome (C-reactive protein).

### Quality assessment

The quality of articles was determined in seven domains containing sequence generation, allocation concealment, reporting bias, performance bias, detection bias, attrition bias, and other potential sources of bias by using the Cochrane Collaboration risk of bias tool. The risk of bias in each domain was classified as low risk, uncertain, and high risk; low-risk items were ≥ 3 and considered as good quality, low-risk items were 2, considered as a fair quality, and if low-risk ≤ 1 considered as poor quality [24]. Any disagreement between the two researchers was resolved in consultation with a third expert researcher (S.M.A).

### Statistical analysis

All concentrations of C-reactive protein (CRP) reported in the studies were calculated in mg/L. Using the method of Hozo et al., 95% CIs, interquartile ranges (IQRs), and standard errors (SEs) reported in the studies were converted to standard deviation (SD) [25]. Weighted mean differences (WMD) and SDs of C-reactive protein (CRP) levels were extracted from the studies. The effect size was determined using the random effect model based on the DerSimonian and Laird overall approach [26]. Cochran’s Q and I2 statistics were used to assess heterogeneity in the results [27]. I^2^ > 40% or *p* < 0.01 represented high heterogeneity among studies [28]. To identify other sources of bias, subgroup analysis was performed based on pre-defined criteria containing country (USA, other countries), participant’s age (≤ 65 and > 65), study duration (x < 12 and x ≥ 12), paricalcitol dosage (x < 2 and x < 2 (µg/d)), BMI of participants (obese and overweight), baseline CRP level (< 5 and x ≥ 5 mg/L).

A sensitivity analysis was performed to identify the impact of each study on the estimated overall effect [29]. Possible publication bias was evaluated by performing Begg’s rank correlation test, Egger’s weighted regression test, and analyzing funnel plots [30, 31].

Fractional polynomial modeling was done to investigate the non-linear effects of paricalcitol dose, intervention duration, and outcome changes [32]. All statistical analyses were performed using STATA version 17 (Stata Corp, College Station, TX). In all analyses performed in the meta-analysis, a p-value < 0.05 was considered statistically significant.

### Certainty assessment

The certainty of the studies included in this review is based on the GRADE guideline (Grading of Recommendations Assessment, Development, and Evaluation) [33], and the certainty quality of the evidence was classified into four categories: high, moderate, low, and very low.

## Results

### Description of studies

**Fig. 1:**
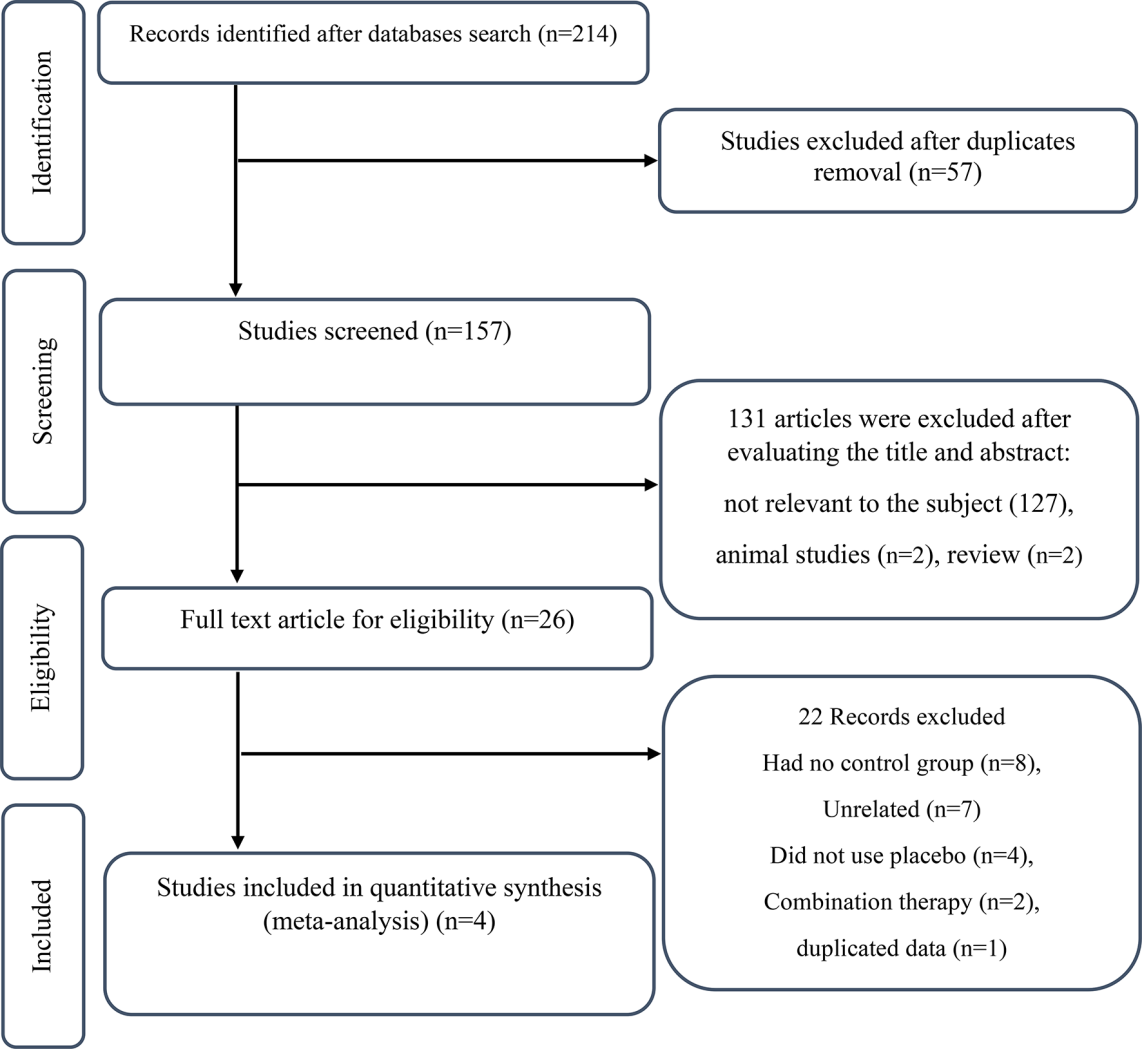
Flowchart of study selection for inclusion of trials in the meta-analysis

In the initial search 182 studies were found, and with the removal of duplicates 60 studies were excluded. By screening the title and abstract of the remaining studies, 99 studies were further excluded. The full text of 26 articles was reviewed, and 22 articles were excluded for the following reasons: had no control group (*n* = 8), unrelated [7], combination therapy [2], did not use placebo [4], and use of duplicated data [1]. Finally, four studies (6 arms) with RCT design were selected for meta-analysis [34–37] (Fig. 1). Had no control group (*n* = 8),

### Study characteristics

Six arms were obtained from 4 studies published from 2008 to 2015, of which 214 patients with CKD (107 intervention group/107 placebo group) were included in this review. Two studies were conducted in the USA [34, 37], 1 study in Sweden [36], and a survey was done in Italy [35]. The intervention duration was from 4 to 12 weeks when the daily dose of paricalcitol supplementation was 1 or 2 (µg/d). In two studies, C-reactive protein levels were investigated in plasma [35, 37], and in one study in serum [34], and there was no report in one other study [36]. Two studies reported the biomarker as CRP [35, 36], and two studies as hs-CRP [34, 37]. The characteristics of the studies included in this review are shown in Table 1.

**Table 1:** Characteristics of the included studies in meta-analysis

studies	Country	Study Design	Participant	Sample size and Sex	Sample size		Intervention Duration (Week)	Means Age		Means BMI		Intervention	
					IG	CG		IG	CG	IG	CG	Paricalcitol dose (µg/d)	Control group
Alborzi et al. 2008(a)	USA	Parallel, R, PC, DB	patients with Chronic Kidney Disease	16 F:14 M:2	8	8	4	72.6 ± 9.1	68.4 ± 12.4	34.3 ± 9.1	35.4 ± 7.7	1	Placebo
Alborzi et al. 2008(b)	USA	Parallel, R, PC, DB	patients with Chronic Kidney Disease	16 F:14 M:2	8	8	4	67.5 ± 9.1	68.4 ± 12.4	35.0 ± 6.5	35.4 ± 7.7	2	Placebo
Lundwall et al. 2015(a)	Sweden	paralell, R, PC, DB	patients with Non-Diabetic Chronic Kidney Disease	24 F:4 M:20	12	12	12	66.1 ± 7.9	59.1 ± 11.6	26.4 ± 3.5	26.8 ± 2.8	1	Placebo
Lundwall et al. 2015(b)	Sweden	paralell, R, PC, DB	patients with Non-Diabetic Chronic Kidney Disease	24 F:NR M:NR	12	12	12	70.8 ± 10.0	59.1 ± 11.6	28.1 ± 2.4	26.8 ± 2.8	2	Placebo
Thethi et al. 2015	USA	paralell, R, PC, DB	patients with type 2 diabetes and chronic kidney disease	46 F:NR M:NR	23	23	12	64 ± 4.5	61 ± 5	NR	NR	1	Placebo
Zoccali et al. 2014	Italy	paralell, R, PC, DB	patients with Chronic Kidney Disease	88 F:31 M:57	44	44	12	63 ± 11	62 ± 12	29 ± 5	29 ± 5	2	Placebo

*Abbreviations* IG, intervention group; CG, control group; DB, double-blinded; SB, single-blinded; PC, placebo-controlled; CO, controlled; RA, randomized; NR, not reported; F, Female; M, Male; NR, not reported

### Risk of bias

An assessment of the risk of bias is presented in Table S2. All the studies included in this meta-analysis had a double-blind randomized control trial design and were of good quality. Two out of four studies reported the details of their randomization [34, 37]. Two studies stated the method of allocation concealment in their review [34, 35]. Selective reporting bias was low in two studies [36, 37] and high in the other two studies [34, 35]. Other sources of bias and blinding (outcome assessment) were unclear in all studies [34–37]. Incomplete outcome data was low risk in all studies [34–37].

### Meta-analysis

**Fig. 2:**
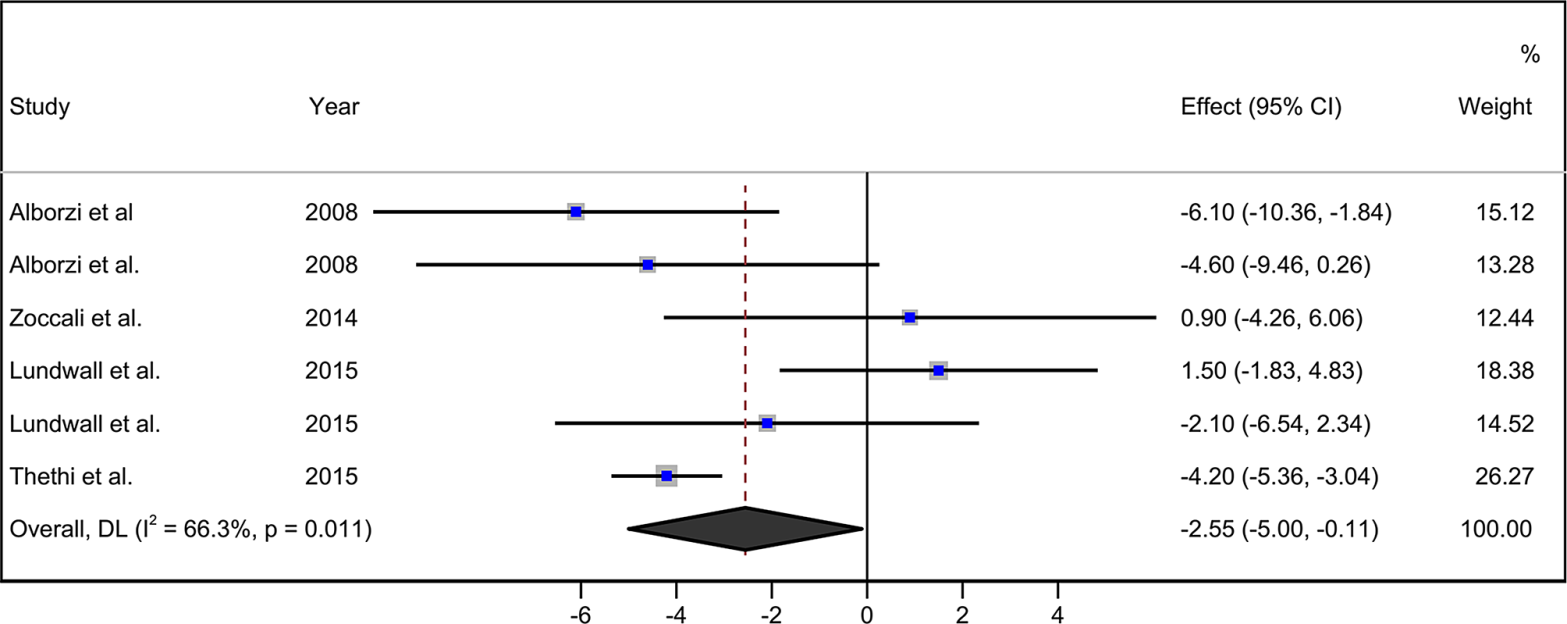
Forest plot detailing weighted mean difference and 95% confidence interval (Cls) for the effect of paricalcitol supplementation on C-reactive protein in CKD patients. *Note * Weights are from random-effects model

The pooled analysis showed that paricalcitol supplementation in CKD patients significantly reduces the level of CRP compared to the control group [WMD = -2.55 mg/L, 95% CI (-4.99 to -0.11), I^2^ = 66.3%] (Fig. 2).

### Subgroup analysis

We performed subgroup analysis based on the CRP level baseline, the dose of paricalcitol supplementation, duration of intervention, country, body mass index (BMI), and age of participants. The subgroup analysis performed showed that there was no significant difference between the categories of age) *p* = 0.91), intervention duration (*p* = 0.08), intervention, paricalcitol dosage (*p* = 0.72), and CRP level at baseline (*p* = 0.65). Subgroup analysis based on the study country showed that in studies conducted in the USA, contrary to studies conducted in other countries, oral paricalcitol supplementation was able to reduce significantly CRP levels in CKD patients (studies conducted in the USA: WMD= -4.34; 95% CI= )-5.43, -3.25(; P = < 0.001; studies conducted in other countries: WMD = 0.35; 95% CI= (-2.01, 2.72); *P* = 0.77). The heterogeneity of studies conducted in countries other than the US was significantly lower (test for heterogeneity: *P* = 0.43 and I^2^ = 0.0%). Subgroup analysis showed that supplementation with paricalcitol in obese people leads to a significant decrease in CRP levels in contrast to that with overweight people. In studies that were conducted on obese people: WMD= -5.44; 95% CI= )-8.64, -2.24 (; *P* = 0.001; and studies in other countries: WMD = 0.35; 95% CI= (-2.01, 2.72); *P* = 0.77. The analysis also showed that in overweight people supplementation with paricalcitol showed significantly reduced heterogeneity (test for heterogeneity: *P* = 0.43 and I^2^ = 0.0%). The details of each subgroups analyses performed are summarized in Table S3.

### Influence analysis

Influence analysis was performed to determine the effect of each on the estimated pooled effect size. The result of removing all of the studies on the effect size ranges from − 1.89 mg/L (95% CI= -4.68, 0.89) to -3.75 mg/L (95% CI=-5.45, -2.05). Removing the articles of Alborzi et al. [34] 2008, Lundwall et al. 2015 [36], and Thi et al. 2015 [37], did not lead to a significant change in CRP levels due to supplementation with paricalcitol.

### Publication bias

Funnel plots showed no effect of selection of publication as the source of bias; moreover, Egger’s test and Begg’s rank correlation test did not show any significant publication bias (P:0.59, and P:0.80 respectively) (Fig. S1).

### Dose-response

Dose-response analysis was done to find a relationship between the dose of paricalcitol supplementation and effect size. No significant relationship between the dose of paricalcitol and effect size was observed (P _dose-response_= 0.1, P _non-linearity_: 0.4), as shown in Figure S2 and Table S5.

### Certainty assessment

A significant limitation was observed only in the imprecision part of the grade photometry tool. Therefore, the certainty of the evidence of the effect of paricalcitol on the CRP level was considered moderate. The details of the assessment of the certainty of the evidence are shown in Table S4.

## Discussion

In this systematic review and meta-analysis, we summarized the evidence of randomized, double-blind, placebo-controlled studies on the effect of oral paricalcitol supplementation on C-reactive protein levels in CKD patients. To the best of our knowledge, this is the first meta-analysis study investigating the impact of oral paricalcitol supplementation on C-reactive protein concentration.

The main finding of this review study shows that supplementation with oral paricalcitol can reduce the level of C-reactive protein compared with the placebo group. BMI and country were two important factors that affected the outcomes of this study. Oral paricalcitol supplementation in obese people, unlike in overweight people, leads to a significant decrease in C-reactive protein (*p* < 0.001 and *p* = 0.77, respectively). Performing the subgroup analysis showed that the pooling of the results of the studies that were conducted in the United States, contrary to the result of studies that were conducted in countries other than the USA, led to a finding of a significant decrease in the levels of C-reactive protein in CKD patients (*p* < 0.001, *p* = 0.77, respectively).

While there is no significant difference between different categories of intervention duration, as the subgroup of effect sizes led to a significant decrease in C-reactive protein on the duration of interventions x < 12 weeks, as opposed to interventions x ≥ 12 weeks (*p* = 0.001, *p* = 0.45 respectively), it seems that the effect of paricalcitol supplementation on C-reactive protein levels decreases after 12 weeks.

In the prospective, open-label, and pilot study conducted by Navarro-González et al. in 2013, on stable hemodialysis patients who had previously consumed calcitriol, oral paricalcitol supplementation for 12 weeks significantly reduced the levels of TNF-a and IL-6 inflammatory biomarkers [38]. Therefore, the result of this meta-analysis was consistent with the outcome of this observational study. In this regard, in the study conducted by Stubbs et al., 2010, on the seven ESRD patients with vitamin D insufficiency, paricalcitol administration did not lead to a significant change in the levels of TNF-a and IL-6 biomarkers [39], although this study did not report CRP changes. In the another studies conduced by Thethi et al. showed that daily receiving 1 mcg paricalcitol orally for 12 weeks had no significant effect on TNF-a and IL-6 levels in patients with type 2 diabetes and chronic kidney disease. The result in this study was therefore contrary to our findings, which could be because the participants in this intervention had received cholecalciferol for eight weeks before receiving paricalcitol. In another study conducted by Moe et al. in 2001, on hemodialysis patients, showed that receiving 4 microgparicalcitol by injection three times in weeks for 12 weeks, did not significantly change the levels of inflammatory markers; however, this study did not report the effect of paricalcitol on C-reactive protein level [40]. The difference between the result of the above study and the findings from our review may be due to several reasons: (1) paricalcitol supplementation in this study was three times a week, while supplementation in the studies used in the meta-analysis of our study was daily; considering that the levels of injected paricalcitol after 24 h minimal compared to the maximum possible amount, this may explain the lack of effect of the supplementary aid. (2) Moe et al.‘s study was conducted on people with lower supplement levels (< 200 pg/ml), while we did not have this precondition in this meta-analysis. (3) Measurement of inflammatory biomarkers in the study by Moe et al. was done ex vivo, whilst, in the studies used in this meta-analysis, inflammatory biomarkers were measured directly from blood samples.

The anti-inflammatory effects of paricalcitol are independent of changes in blood pressure, GFR, and PTH, so its anti-inflammatory effects are considered to be related to non-hemodynamic mechanisms and not related to levels of PTH [34]. In a study conducted by Navarro-González et al. (2013), it was shown that supplementation with paricalcitol leads to the improvement of the expression pattern of inflammatory genes [38]. In an intervention study performed on mice, it was shown that paricalcitol reduces the activity of T cells and macrophages by blocking NF-κB activation [41]. CRP has been proposed to influence the pathogenesis of atherosclerosis by some mechanisms including: (1) activation of the complement system after interaction with damaged cells and (2) stimulation of tissue factor secretion from monocytes, which increases other inflammatory mediators. CRP plays a role in calcium-dependent conditions in vitro, which leads to an increase in the binding of very low-density lipoprotein (VLDL) and low-density lipoprotein (LDL). Paricalcitol supplementation may therefore reduce the risk of atherosclerosis in CKD patients by decreasing CRP levels [9].

Regarding the clinical application of the results of this meta-analysis, we detected a significant reducing effect on CRP, as a widely accepted marker of systemic inflammation, following paricalcitol intervention. This effect size is higher than the changes in the minimum clinically important difference (MCID) considered by clinicians in practice. The MCID changes for the CRP variable based on previous studies is 0.5 mg/L [42, 43], whilst in the present study the effect size is -2.5 mg/L. Based on the review of Bazley et al., CRP level has a strong relationship with 1- year mortality in hemodialysis patients and is used as a strong predictor of mortality. It is suggested to investigate the effect of paricalcitol supplementation on mortality in CKD patients in future studies. Furthermore, in the study conducted by Nata et al. on hemodialysis patients with vitamin D deficiency, showed that high-dose ergocalciferol supplementation (in patients with serum 25(OH)D levels from 20 to 29.9 ng/ml: 100,000 units monthly for 2 months, and in patients with less than 20 ng/ml: 100 000 units weekly for 2 months) had a greater reduction effect on IL-6 than supplementation with conventional dosage (in patients with serum 25(OH)D levels from 20 to 29.9 ng/ml: 50,000 units monthly for 2 months, and in patients with less than 20 ng/ml: 50,000 units weekly for 2 months). Based on these results, it is suggested to conduct future trials to compare paricacitol supplementation effectiveness in different dosages and patients with different baseline serum 25[OH]D [44].

The strengths of this study include consideration of all heterogeneity groups by performing subgroup analyses, the performance of dose-response analyses, ascertainment of evidence, and the finding of non-significant publication bias. Limitations of this study are the sample size and short duration of the study, the observed serious limitation of effect, the existence of different types of C-reactive protein (CRP and hs-CRP), and the different samples tested in various studies (serum and plasma).

## Conclusion

In conclusion, the findings of this meta-analysis revealed that supplementation with oral paricalcitol can significantly reduce C-reactive protein in CKD patients. Also, this review showed that paricalcitol supplementation has the necessary potential for further research in the future to reach a definitive conclusion about its effectiveness. Well-designed clinical trials with larger sample sizes are needed to confirm this result and the other anti-inflammatory effects of oral paricalcitol supplementation in various groups of patients.

### Electronic supplementary material

Below is the link to the electronic supplementary material.

Supplementary Material

## Data Availability

The datasets generated and/or analysed during the current study are not publicly available but are available from the corresponding author on reasonable request.
