# Supplementary material for: The effect of oral supplementation of Paricalcitol on C-reactive protein levels in chronic kidney disease patients: GRADE-assessed systematic review and dose-response meta-analysis of data from randomized controlled trials
Source: BMC Pharmacol Toxicol. 2024 Feb 23;25:19. doi: 10.1186/s40360-024-00740-y (PMC10885610; doi:10.1186/s40360-024-00740-y)
Supplement: Supplementary file 1 — Supplementary Material [file 40360_2024_740_MOESM1_ESM.docx]

**The effect of oral supplementation of Paricalcitol on C-reactive protein levels in chronic kidney disease patients: GRADE-assessed systematic review and dose-response meta-analysis of data from randomized controlled trials**

Electronic Supplementary Material Appendix Tables S1-S5, Appendix Figures S1-S2, and Electronic Supplementary Material References

**Electronic Supplementary Material** **Appendix Table S1**. Search strategy to find potential eligible trials for inclusion in the meta-analysis of Paricalcitol supplementation and CRP level.

| **PubMed:** 34 results**,** 2/4/2023 |
| --- |
| (((((Paricalcitol[Title/Abstract]) OR (Paricalcitol[Supplementary Concept]) OR (Zemplar[Title/Abstract])) OR (1,25-dihydroxyergocalciferol[Title/Abstract]) OR (1,25-dihydroxyergocalciferol[Supplementary Concept]) OR (19-nor-1,25-(OH)2-vitamin D2[Title/Abstract]) OR (19-nor-1,25-(OH)2-vitamin D2[Title/Abstract]) OR (19-nor-1alpha,25-dihydroxyvitamin D2[Title/Abstract]) OR (19-nor-1,25-(OH)2D2[Title/Abstract])) AND (((Paricalcitol[Title/Abstract]) OR (Paricalcitol[Supplementary Concept]) OR (Zemplar[Title/Abstract])) OR (1,25-dihydroxyergocalciferol[Title/Abstract]) OR (1,25-dihydroxyergocalciferol[Supplementary Concept]) OR (19-nor-1,25-(OH)2-vitamin D2[Title/Abstract]) OR (19-nor-1,25-(OH)2-vitamin D2[Title/Abstract]) OR (19-nor-1alpha,25-dihydroxyvitamin D2[Title/Abstract]) OR (19-nor-1,25-(OH)2D2[Title/Abstract]))) AND ((((((("inflammation"[Title/Abstract]) OR ("c-reactive protein"[Title/Abstract])) OR ("CRP"[Title/Abstract])) OR ("inflammation"[MeSH Major Topic])) OR ("c-reactive protein"[MeSH Major Topic])) OR ("Hs-CRP"[Title/Abstract])) OR ("High sensitivity C-reactive protein"[Title/Abstract]))) AND (((((((((randomized[Title/Abstract]) OR (placebo[Title/Abstract])) OR (clinical trials[Title/Abstract])) OR (randomly[Title/Abstract])) OR (trial[Title/Abstract])) OR (randomized controlled trial[Title/Abstract])) OR (RCT[Title/Abstract])) OR (("Clinical Trials as Topic"[Mesh]) OR ( "Clinical Trial" [Publication Type] OR "Controlled Clinical Trial" [Publication Type] )))) |
| **Scopus**: 131, 2/4/2023  ( ( TITLE-ABS-KEY ( paricalcitol )  OR  TITLE-ABS-KEY ( zemplar )  OR  TITLE-ABS-KEY ( "1,25-dihydroxyergocalciferol" )  OR  TITLE-ABS-KEY ( "19-nor-1,25-(OH)2-vitamin D2" )  OR    TITLE-ABS-KEY ( "19-nor-1alpha,25-dihydroxyvitamin D2" )  OR  TITLE-ABS-KEY ( "19-nor-1,25-(OH)2D2" ) ) )  AND  ( ( TITLE-ABS-KEY ( "inflammation" )  OR  TITLE-ABS-KEY ( "c-reactive protein" )  OR  TITLE-ABS-KEY ( "CRP" )  OR  TITLE-ABS-KEY ( "Hs-CRP" )  OR  TITLE-ABS-KEY ( "High sensitivity C-reactive protein" ) ) )  AND  ( ( TITLE-ABS-KEY ( randomized )  OR  TITLE-ABS-KEY ( placebo )  OR  TITLE-ABS-KEY ( clinical  AND  trials )  OR  TITLE-ABS-KEY ( randomly )  OR  TITLE-ABS-KEY ( trial )  OR  TITLE-ABS-KEY ( randomized  AND  controlled  AND  trial )  OR  TITLE-ABS-KEY ( rct ) ) ) |
| **Web of Sciences**: 51, 2/4/2023  Query #1  **((((((TS=("Paricalcitol")) OR TS=("Zemplar")) OR TS=("1,25-dihydroxyergocalciferol")) OR TS=("19-nor-1,25-(OH)2-vitamin D2")) OR TS=("19-nor-1alpha,25-dihydroxyvitamin D2")) OR TS=(19-nor-1,25-(OH)2-vitamin D2)**  Edit  Query #2  **((((TS=("inflammation")) OR TS=("c-reactive protein")) OR TS=("CRP")) OR TS=("Hs-CRP")) OR TS=("High sensitivity C-reactive protein")**  Edit  Query #3  **((((((TS=(randomized)) OR TS=("placebo")) OR TS=("clinical trials")) OR TS=("randomly")) OR TS=("trial")) OR TS=("randomized controlled trial")) OR TS=("RCT")**  **#1 AND #2 AND #3** |
| **Total**: 214 |

| **Study** | **Random sequence generation** | **Allocation concealment** | **Selective reporting** | **Other sources of bias** | **Blinding (participants and personnel)** | **Blinding (outcome assessment)** | **Incomplete outcome data** | **General risk of bias** |
| --- | --- | --- | --- | --- | --- | --- | --- | --- |
| Alborzi et al. 2008 | L | L | H | U | L | U | L | Low |
| Lundwall et al.2015 | U | U | L | U | L | U | L | Low |
| Thethi et al.2015 | L | U | L | U | L | U | L | Low |
| Zoccali et al.2014 | U | L | H | U | L | U | L | Low |

**Electronic Supplementary Material** **Appendix Table S2.** Quality of trials included in the meta-analysis of Paricalcitol supplementation and CRP levels.

Legend: General Low risk < 2 high risk; General moderate risk = high risk; General high risk > 2 high risk

**Electronic Supplementary Material** **Appendix Table S3**. Subgroup analyses of Paricalcitol supplementation in adults.

|  | NO | WMD (95%CI) | P-value | heterogeneity | | |
| --- | --- | --- | --- | --- | --- | --- |
|  |  |  |  | P heterogeneity | I^2^ | P between sub-groups |
| Subgroup analyses of Paricalcitol supplementation on CRP level | | | | | |  |
| Overall effect | 6 | -2.554.99-) , -0.11) | **0.04** | **0.01** | 66.3% |  |
| **Intervention duration (weeks)** | | | | | | |
| x<12 | 2 | -5.44 (-8.64, -2.24) | **0.001** | 0.64 | 0.0% | 0.08 |
| x≥12 | 4 | -1.29 (-4.66, 2.07) | 0.45 | **0.004** | 77.1% |  |
| **Intervention dose (µg/d)** | | | | | | |
| x<2 | 3 | -2.90 (-6.80, 0.99) | 0.14 | **0.004** | 82.3% | 0.72 |
| x≥2 | 3 | -2.03 (-5.01, 0.94) | 0.18 | 0.31 | 13.6% |  |
| Mean age | | | | | | |
| x>65 | 4 | -2.62 (-6.22, 0.97) | 0.15 | **0.03** | 66.3% | 0.91 |
| x≤65 | 2 | -2.29 (-7.13, 2.54) | 0.35 | 0.05 | 72.0% |  |
| **Trial location** | | | | | | |
| USA | 3 | -4.34 (-5.43, -3.25) | **<0.001** | 0.69 | 0.0% | **<0.001** |
| Others | 3 | 0.35 (-2.01, 2.72) | 0.77 | 0.43 | 0.0% |  |
| **Baseline BMI (kg/m^2^)** |  | | | | | |
| Obese (30-34.9) | 2 | -5.44 (-8.64, -2.24) | **0.001** | 0.64 | 0.0% | **0.004** |
| Overweight (25-29.9) | 3 | 0.35 (-2.01, 2.72) | **0.77** | 0.43 | 0.0% |  |
| **CRP in baseline (mg/L)** | | | | | | |
| x<5 | 3 | -2.88 (-7.89, 2.13) | 0.26 | **0.01** | 77.5% | 0.65 |
| x≥5 | 2 | -4.06 (-5.18, -2.94) | **<0.001** | 0.37 | 0.0% |  |

Abbreviations: **CI**, confidence interval; **WMD**, weighted mean differences; **D**, double-blind; **S**, single-blind; **B**, both; **M**, male; **EPA**, Eicosapentaenoic acid; **DHA**, Docosahexaenoic acid

**Electronic Supplementary Material** **Appendix Table S4.** GRADE evidence table for Paricalcitol compared to placebo for endothelial functions (FMD).

| **Certainty assessment** | | | | | | | **№ of patients** | | **Effect** | | **Certainty** | **Importance** |
| --- | --- | --- | --- | --- | --- | --- | --- | --- | --- | --- | --- | --- |
| **№ of studies** | **Study design** | **Risk of bias** | **Inconsistency** | **Indirectness** | **Imprecision** | **Other considerations** | **Paricalcitol** | **placebo** | **Relative (95% CI)** | **Absolute (95% CI)** |  |  |
| **New outcome** | | | | | | | | | | | | |
| 6 | randomized trials | not serious | serious^a^ | not serious | serious^b^ | dose-response gradient | 107 | 107 | - | MD **2.55 mg/L lower** (4.99 lower to 0.11 lower) | ⨁⨁⨁◯ Moderate | IMPORTANT |

CI: confidence interval; MD: mean difference

**Explanations**

a. Serious inconsistency since I^2^=66.3%. Downgrade

b. The optimal information size was not sufficient, downgraded.

**Table S5.** The effects of different doses of Paricalcitol supplementation on CRP from the non-linear dose-response meta-analysis (mean difference and 95% confidence interval).

| 2.5 Mcg | 2 Mcg | 1 Mcg | 0  (Ref) | **Paricalcitol dosage (Mcg/d)** |
| --- | --- | --- | --- | --- |
| -1.6  (-6.4– 3.2) | -2.04  (-4.9– 0.8) | -2.9  (-7.1– 1.3) | 0 | FMD |

**Abbreviations: CRP;** C-reactive protein

**Electronic Supplementary Material** **Appendix Figure S1**. Funnel plot of the effects of Paricalcitol supplementation on CRP levels.


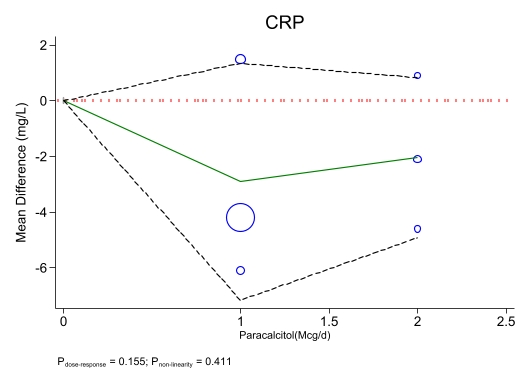


**Electronic Supplementary Material** **Appendix Figure S2.** Dose-dependent effect of Paricalcitol supplementation treatment on CRP levels.
